# Chemical Constituents and Antimicrobial and Antioxidant Activities of Essential Oil from Dried Seeds of *Xylopia aethiopica*

**DOI:** 10.1155/2024/3923479

**Published:** 2024-02-14

**Authors:** Samba Fama Ndoye, Yoro Tine, Insa Seck, Lalla Aicha Ba, Seydou Ka, Ismaila Ciss, Abda Ba, Seynabou Sokhna, Moussa Ndao, Rokhaya Sylla Gueye, Nango Gaye, Abdoulaye Diop, Jean Costa, Julien Paolini, Matar Seck

**Affiliations:** ^1^Laboratoire de Chimie de Coordination Organique (LCCO), Département de Chimie, Faculté des Sciences et Techniques, Université Cheikh Anta Diop de Dakar, BP 5005, Dakar-Fann, Senegal; ^2^Laboratoire de Chimie Organique et Thérapeutique, Faculté de Médecine, de Pharmacie et d'Odontologie de l'Université Cheikh Anta Diop de Dakar, BP 5005, Dakar-Fann, Senegal; ^3^Université Amadou Mahtar MBOW, BP 45927 Dakar Nafa VDN, Dakar-Fann, Senegal; ^4^Laboratoire Bactériologie-Virologie, CHU Aristide Le Dantec, Université Cheikh Anta Diop de Dakar, BP 5005, Dakar-Fann, Senegal; ^5^Université de Corse, UMR CNRS 6134 SPE, Laboratoire de Chimie des Produits Naturels, Campus Grimaldi, BP 52, Corte F-20250, France

## Abstract

The study aimed to investigate the chemical composition and antimicrobial and antioxidant activities of the essential oil from dried seeds of *Xylopia aethiopica*. The essential oil was obtained by hydrodistillation and analyzed by GC/FID and GC/MS. The essential oil yield was 1.35%. Forty-nine compounds were identified in the essential oil with 1,8-cineole (16.3%), *β*-pinene (14.8%), *trans*-pinocarveol (9.1%), myrtenol (8.3%), *α*-pinene (5.9%), and terpinen-4-ol (5.6%) as major components. The antimicrobial activity of this essential oil was studied using disk diffusion and broth microdilution methods on four bacteria (*Staphylococcus aureus, Enterococcus faecalis, Escherichia coli,* and *Pseudomonas aeruginosa*) and one fungus *(Candida albicans)*. The essential oil exhibited excellent activity against *S. aureus*, *E. faecalis*, and *C. albicans* and moderate activity against *E. coli*. Among all strains tested, *C. albicans* showed the best sensitivity with a MIC of 50 mg/mL. The antioxidant activity was examined using a DPPH-free radical scavenging assay. The essential oil of *X. aethiopica* showed low antioxidant activity (IC_50_ = 784.604 ± 0.320 mg/mL) compared to that of ascorbic acid and the reference compound (IC_50_ = 0.163 ± 0.003 mg/mL). The results indicate that consumption of *X. aethiopica* seeds can reduce the virulence of food-borne pathogens and their resistance to antibiotics.

## 1. Introduction

Free radicals and other reactive oxygen species, which are highly reactive compounds, are produced naturally in the cell and play a key physiological role. However, these unstable compounds can exert negative effects on the immune system if their production is greater than that of antioxidants, which are able to neutralize or scavenge free radicals. Overproduction of free radicals known as oxidative stress is involved in the development of several diseases such as cancer, cardiovascular disease, and Alzheimer's or Parkinson's diseases or in cell ageing [1, 2]. On the other hand, the proliferation of resistance of bacteria to the available antibiotics exacerbates the damage caused by infectious diseases [3]. In 2011, the WHO (World Health Organization) called for intensified research into new antibacterial drugs to deal with this scourge, but only a few new molecules are under development [4, 5]. Consequently, there has been considerable and growing interest in identifying new sources of antioxidant and antimicrobial potential from natural, safe, and inexpensive sources [6].

*Xylopia aethiopica,* commonly known as “Ethiopian pepper” or “Guinea pepper,” is an aromatic tree that belongs to the family of Annonaceae. This plant, native to lowland rainforest and moist fringe forest in the savanna zones of Africa, is largely found in West, Central, and Southern Africa. It is a small tree which can reach 20 m in height [7]. Its leaves are simple, alternate, oblong, elliptic, or ovate. *X. aethiopica* has bisexual, creamy-green flowers that are either solitary or in clusters of 3 to 5-odd, sinuous, branched spikes, or cymes. The fruits of *X. aethiopica* are in the form of twisted bean pods which are dark brown, cylindrical, 2.5 to 5 cm long, and 4 to 6 mm thick [8]. The matured fruits are usually green but become brownish-black after drying. The fruit which is a popular spice in Senegal is widely used in the preparation of a local coffee called “Café Touba” [9]. It is widely used in traditional medicine in the treatment of cough, dysentery, edema, rheumatism stomachache, dizziness, amenorrhea, lumbago, and neuralgia [10–13]. Some studies have also demonstrated the efficacy of *X. aethiopica* oil as anticancer [14–16], antioxidant [7, 12, 17–20], antimicrobial [13, 19, 21–30], anti-inflammatory [18, 22, 31], anticholinesterase [7], antimalarial [13], antitrypanosomal [13], antidepressant [32], and insecticidal [33]. The chemical composition of the essential oil of *X. aethiopica* fruits is very diverse. Nine chemotypes have been reported, and this includes *β*-pinene [14, 19, 25, 31, 34–36], terpinen-4-ol [7, 37], 1,8-cineole [13, 38–40], germacrene D [17], eugenol [20], sabinene [30, 41–43], 4-isopropylbenzyl alcohol [44], 3-carene [45], and santalol [46]. To our knowledge, only one study on the chemical composition of essential oils of *X. aethiopica* seeds from Senegal has been reported. The essential oils were mainly dominated by *β*-pinene, 1,8-cineole, and *α*-pinene in variable proportions [47]. Thus, the aim of this study was to characterize the chemical profile and the antimicrobial and antioxidant activities of the essential oil of *X. aethiopica* seeds.

## 2. Materials and Methods

### 2.1. Plant Material

Seeds of *X. aethiopica* were purchased from Tilène market in Dakar. They were authenticated in the Laboratory of Pharmacognosy, Faculty of Medicine, Pharmacy and Odontology of Cheikh Anta Diop University in Dakar (Senegal).

### 2.2. Essential Oil Extraction

The seeds were powdered using a Brabender brand mechanical grinder. To extract the essential oil, the sample was hydrodistilled (4 h) using a Clevenger-type apparatus according to the method recommended in the European Pharmacopoeia [48]. After hydrodistillation, the essential oil collected was stored at 4°C before analyses. The extraction yield of essential oil was calculated as a percentage (w/w) based on the weight of the dried seeds.

### 2.3. Physical Characteristics of Essential Oils

Physical examinations regarding density, refractive index, and optical rotation of essential oils were carried out in this work.

#### 2.3.1. Density of Essential Oil

The gravimetric method measured the density of essential oil. The weight of the empty pycnometer on an analytical balance was denoted (*P*_0_). Besides, the filled pycnometer with distilled water was weighted (*P*_1_), and that of the pycnometer filled with the oil of *X. aethiopica* was coded (*P*_2_). The following formula was used to calculate the density of the volatile oils:

(1) $$\begin{array}{c} D=\frac{P_{2}-P_{0}}{P_{1}-P_{0}}. \end{array}$$

#### 2.3.2. Refractive Index Measurement

A refractometer (Atago) with sodium lamp at 589 nm at room temperature 25°C was used to measure the refractive index. The sample was injected into the prism of the instrument using a pipette.

#### 2.3.3. Determination of Optical Rotation

Polaritonic E polarimeter measured the optical rotation, which measured the degree of rotation. The zero point of the polarimeter was adjusted and determined. A solution of 59.8 g/L of the essential oil sample was prepared in analytical grade methanol, and the reading was made with 1 dm tube at 25°C.

### 2.4. GC and GC/MS Analysis

The chromatographic analyses were carried out using a *Perkin–Elmer Autosystem XL* GC apparatus (*Waltham*, MA, USA) equipped with dual flame ionization detection (FID) system and fused-silica capillary columns, namely, Rtx-1 (polydimethylsiloxane) and Rtx-wax (polyethylene glycol) (60 m × 0.22 mm i.d; film thickness 0.25 *μ*m). The oven temperature was programmed from 60 to 230°C at 2°C/min and then held isothermally at 230°C for 35 min: hydrogen was used as carrier gas (1 mL/min). The injector and detector temperatures were maintained at 280°C, and samples were injected (0.2 *μ*L of pure oil) in the split mode (1 : 50). Retention indices (RI) of compounds were determined relative to the retention times of a series of n-alkanes (C_5_–C_30_) by linear interpolation using the equation of Van den Dool and Kratz (1963) through Perkin–Elmer software (total Chrom navigator). The relative percentages of the oil constituents were calculated from the GC peak areas, without the application of correction factors.

Samples were also analyzed with a Perkin–Elmer Turbo mass detector (quadrupole) coupled to a *Perkin–Elmer Autosystem XL*, equipped with Rtx-1 and Rtx-wax fused-silica capillary columns. The oven temperature was programmed from 60 to 230°C at 2°C/min and then held isothermally at 230°C (35 min): hydrogen was used as carrier gas (1 mL/min). The following chromatographic conditions were employed: injection volume, 0.2 *μ*L of pure oil; injector temperature, 280°C; split, 1 : 80; ion source temperature, 150°C; ionization energy, 70 eV; MS (EI) acquired over the mass range, 35–350 da; scan rate, 1 s.

The identification of the components was based on (a) the comparison of their GC retention indices (RI) on nonpolar and polar columns, determined from the retention times of a series of n-alkanes with linear interpolation, with those of authentic compounds or literature data; (b) computer matching with commercial mass spectral libraries [49–51] and comparison of spectra with those of our personal library; and (c) comparison of RI and MS spectral data of authentic compounds or literature data.

### 2.5. Microbial Strains

Antibacterial activity of the essential oils of *X. aethiopica* was carried out by using five pathogenic strains: *Staphylococcus aureus ATCC 29213, Enterococcus faecalis ATCC 29212, Escherichia coli ATCC 35218, Pseudomonas aeruginosa ATCC27853, and Candida albicans ATCC 24433*. All of the strains were grown on Mueller–Hinton agar for the bacteria and Sabouraud dextrose agar with chloramphenicol for yeast.

### 2.6. Determination of Antibacterial Activity

The sensibility of the five pathogenic strains to the essential oils was assayed using the agar disc diffusion method [52]. Inocula were prepared by diluting overnight cultures in Mueller–Hinton broth (*MHB*; Oxoid) medium to approximately 10^8^ CFU/mL. Filter paper discs (*Whatman* disc, 6 mm diameter) were impregnated with 25 *μ*L of the essential oil and placed onto the inoculated Petri dishes containing Mueller–Hinton 2 agar. After incubation at 37 ± 1°C for 24 h for bacteria, the diameters of inhibition zones were measured (mm) and recorded as the mean ± standard deviation. Each test was performed in triplicate separate. According to the width of the inhibition zone diameter expressed in mm, results were appreciated as follows: not sensitive (−) for diameter equal to or below 8.0 mm, moderately sensitive (+) for diameter between 8.0 and 14.0 mm, sensitive (++) for diameter between 14.0 and 20.0 mm, and extremely sensitive (+++) for diameter equal to or longer than 20.0 mm.

For the determination of the minimum inhibitory concentration (MIC), which represents the concentration that completely inhibits the growth of microorganisms, a microdilution broth susceptibility assay was used, as recommended by the National Committee for Clinical Laboratory Standards [53]. All tests were performed in MHB supplemented with Tween 80 detergent to a final concentration of 0.5% (v/v). Dilutions series were prepared from 3.125 to 100.0 mg/mL of the oil in a 96-well microtiter plate. 160 *μ*L of MHB was added onto microplates and 20 *μ*L of tested solution. Then, 20 *μ*L of 1 × 10^8^ CFU/mL of standard microorganism suspension was inoculated onto microplates. Plates were incubated at 37°C for 24 h. The same test was performed simultaneously for the growth control (MHB + Tween 80) and sterility control (MHB + Tween 80 + test oil). The MIC is defined as the lowest concentration of the samples at which the bacterium does not demonstrate visible growth. The bacterium growth was indicated by turbidity.

### 2.7. Antioxidant Activity

The antiradical activity of the essential oil of *X. aethiopica* was estimated by way of the 2,2′-diphenyl-1-picrylhydrazyl (DPPH) test according to the method described by Scherer *et al* [54]. Then, the DPPH^.^ solution was prepared by dissolving 4 mg of DPPH radical in 100 mL of methanol, and the solution was stirred in the dark for 1 hour. Ascorbic acid was used as standard. About 0.1 mL aliquots of methanolic solution of the sample or standard at different concentrations were each added to 3.9 mL of a DPPH^.^ methanolic solution. After homogenization, the mixture was incubated in the dark at room temperature for 30 minutes, and the absorbance was measured at 517 nm. The blank sample consisted of 0.1 mL of methanol added to 3.9 mL of DPPH^.^. Analyses were performed in triplicate. The radical scavenging activity was expressed as PI=[(*A*_0_  −  *A*_1_)/*A*_0_] × 100, where PI was the percentage inhibition, *A*_0_ was the absorbance of the blank, and A_1_, the absorbance in the presence of the sample or standard at different concentrations. The results were also indicated as IC_50_ (the concentration of sample required to scavenge 50% of DPPH radicals).

## 3. Results and Discussion

### 3.1. Yield and Physical Properties

The volatile oil of *X. aethiopica* has a yellow-pale color, and the percentage yield of essential oil obtained from the hydrodistillation method was 1.35%. This result is comparable to those previously obtained by Thiam et al. in Senegal (1.2%) [47] and Usman et al. in Nigeria (1.3%) [23]. This extraction yield was higher than those reported by two different studies from Cameroon (0.56% and 0.60%) [25, 55], one from Nigeria (0.42%) [40], and one from Benin (0.80%) [41]. However, it was lower than most extraction yields reported in the literature (2.18–5.24%) [7, 16, 17, 19, 27, 31, 35, 36, 45, 56].

Besides, physical parameters, which are useful for the quality of essential oils, were determined. Then, a density of 0.902 g·cm^−3^ was found, allowing the oil to be above the water during extraction. It is comparable to that found by Vyry Wouatsa et al. which was 0.9239 [25]. *Xylopia*'*s* essential oils show a refractive index of 1.334 and an optical rotation of +7.69°. These physical properties are also comparable to those of Vyry Wouatsa et al. which established a refractive index of 1.488, an optical rotation of +6.133, and a density of 0.9239 (Table 1) [25].

### 3.2. Chemical Composition

The analysis of the essential oil by GC/FID and GC/MS allowed the identification of 49 compounds accounting for 95.5% of the total composition (Table 2). Forty-eight compounds were identified by comparing their electronic impact-mass spectra and their retention indices (RI) with those of the laboratory-made library. One constituent (with an asterisk in Table 2) was identified by comparison of their EI (electron ionization) mass spectra and their nonpolar RI with those of commercial libraries. The volatile oil displayed oxygenated monoterpenes (64.7%) and hydrocarbon monoterpenes (29.3%) rich oil. Indeed, seed essential oils exhibited 1,8-cineole (16.4%), *β*-pinene (14.8%), trans-pinocarveol (9.1%), myrtenol (8.3%), *α*-pinene (5.9%), terpinen-4-ol (5.6%), and myrtenal (5.2%) as main components.

The chemical composition reported by our study has the same chemical profile as that reported by Thiam et al. but quantitatively different (*β*-pinene 14,8 vs. 31.2%, 1,8-cineole 16,4 vs. 15.1%, *α*-pinene 11.0 vs. 5,9%, sabinene 1.9 vs. 5.0%, trans-pinocarveol 9,1 vs. 4.4%, myrtenol 8.3 vs. 3.9%) [47]. On the other hand, this chemotype combining 1,8-cineole and *β*-pinene has been reported by several authors from different origins (Cameroon [39], Nigeria [13], and Congo Brazzaville [56]). *β*-Pinene has been described as the primary compound in several chemotypes [14, 19, 25, 31, 34–36]. Finally, trans-pinocarveol [27, 35], myrtenol [27], *α*-pinene [19, 36, 44], and terpinen-4-ol [7, 37] have been reported in a certain number of studies. Germacrene D [17], eugenol [20], sabinene [30, 41–43], 4-isopropylbenzyl alcohol [44], 3-carene [45], and santalol [46] were found in certain essential oils of *X. aethiopica* fruits at significant levels while in our sample, they were present in low or no levels. This chemical variability may be due to climatic conditions, soil conditions, and genetic mutations.

### 3.3. Antimicrobial Activity

For the sensitivity test, undiluted essential oil was evaluated on five microbial strains including *Staphylococcus aureus, Enterococcus faecalis, Escherichia coli, Pseudomonas aeruginosa, and Candida albicans* by using the disc diffusion method in agar medium. The essential of *X. aethiopica* exhibited excellent activity against *S. aureus* ATCC 29213, *E. faecalis* ATCC 29212, and *C. albicans* (IZ = 33.4 ± 1.8, IZ = 21.9 ± 1.2 and IZ = 22.1 ± 0.7, respectively) and moderate activity against *E. coli* ATCC 25922 (IZ = 11.3 ± 0.5). However, *P. aeruginosa* ATCC 27853 was not sensitive to the essential (Table 3).

The minimum inhibitory concentration (MIC) was measured to determine the minimum concentration of oil that inhibited the growth of the microbes used in this study. The MIC helps determine the level of resistance of a particular bacterial strain. Several concentrations were prepared and evaluated only on four strains of three bacteria (*E. coli, S. aureus,* and *E. faecalis*) and one fungus (*C. albicans*) because *P. aeruginosa* was not sensitive to the essential oil. Among all strains tested, *C. albicans* showed the best sensitivity, and the MIC measured for the essential oil of *X. aethiopica* was 50 mg/mL (Table 3). However, *E. coli and E. faecalis* are less sensitive to *X. aethiopica* essential oil (MIC > 100 mg/mL).

The good antimicrobial activity of *X. aethiopica* fruit essential oil corroborates previously reported data [13, 19, 21–30]. In addition, a number of studies have shown that the antimicrobial activity of an essential oil is closely linked to its main constituents and their interactions with certain minor constituents [57, 58]. Previously, several studies have demonstrated the antimicrobial properties of essential oils rich in 1,8-cineole against a wide range of microorganisms [59, 60]. 1,8-cineole has been shown to change the shape and size of the bacterial cell (for both Gram-negative and Gram-positive bacteria) [61]. Tegang et al. attributed the antimicrobial activity of *X. aethiopica* essential oil to its high content of bioactive compounds such as *β*-pinene (32.16%) and *α*-pinene (7.39%) which have been isolated, purified, and studied extensively for their antimicrobial activity [19].

### 3.4. Antioxidant Activity

The DPPH radical scavenging method was adopted to assess the antioxidant effect of *X. aethiopica* essential oil, and ascorbic acid was used as a positive control. Based on our results, *Xylopia aethiopica* exhibited antioxidant activity by scavenging DPPH-free radicals. Thus, the IC_50_ value of the essential oil of *Xylopia aethiopica* was 784.604 ± 0.320 mg/mL. However, this activity is very weak compared to that of the reference ascorbic acid (IC_50_ = 0.163 ± 0.003 mg/mL). Besides, chromatographic analysis revealed that the main compound of *Xylopia* essential oil had a maximum of one hydroxyl group. Thus, this low antioxidant activity of volatile compounds could be due to their low capacity to donate an electron or a hydrogen atom to reduce the DPPH radical. Finally, the complex cluster of organic products in the essential oils and the significant amount of monoterpene hydrocarbons could have an antagonistic activity and thus reduce the antioxidant activity. This same remark was made by Alitonou et al. [18] and Tegang et al. [19] who reported low antioxidant activity of *X. aethiopica* essential oils mainly dominated by hydrocarbon compounds. They concluded that the activity observed could be due to the presence of one (or more) minority constituents in the essential oil.

## 4. Conclusion

This study reported the chemical composition and the antibacterial and antioxidant activities of the essential oil from dried seeds of *Xylopia aethiopica*. This essential oil mainly consisted of 1,8-cineole, *β*-pinene, *trans*-pinocarveol, myrtenol, *α*-pinene, and terpinen-4-ol showed excellent activity against *S. aureus*, *E. faecalis*, and *C. albicans* and moderate activity against *E. coli*. However, it had low antioxidant activity. It may have potential applications in food and pharmaceutical products.

## Figures and Tables

**Table 1:** Yield and physical properties of *Xylopia's* essential oil.

Parameters	Essential oils
Extraction yield (%)	1.35
Density (g·cm^−3^)	0.902 ± 0.025
Refractive index	1.334 ± 0.041
Optical rotation (°)	+7.69 ± 0.023 (*c* = 5.98, MeOH)

**Table 2:** Chemical composition of essential oil from the dried seeds of *X. aethiopica*.

*N* ^a^	Compounds	RI *l*^b^	RI *a*^c^	(%)
1	Hexanal	767	768	0.1
2	*α*-Thujene	932	920	0.6
3	*α*-Pinene	936	931	**5.9**
4	Camphene	950	941	0.2
5	Thuja-2,4(10)-diene	946	944	0.4
6	Sabinene	973	963	1.9
7	*β*-Pinene	978	970	**14.8**
8	Dehydro-1,8-cineole	993	977	0.1
9	*α*-Phellandrene	1002	996	0.1
10	*α*-Terpinene	1013	1010	0.9
11	*m*-Cymene	1013	1011	1.6
12	Limonene	1025	1021	1.0
13	1,8-Cineole	1024	1021	**16.4**
14	*Cis*-*β*-ocimene	1029	1025	tr
15	*γ*-Terpinene	1051	1048	1.4
16	*Trans*-sabinene hydrate	1053	1051	0.5
17	(*E*)-linalool oxide (THF)	1058	1056	0.4
18	Terpinolene	1082	1080	0.5
19	*Cis*-sabinene hydrate	1082	1081	0.4
20	Linalool	1086	1086	3.0
21	*α*-Thujone	1089	1089	0.1
22	*α*-Campholenal	1105	1106	0.5
23	Nopinone	1116	1114	2.8
24	Camphor	1123	1122	0.1
25	*Trans*-pinocarveol	1126	1130	**9.1**
26	Sabinene ketone	1132	1131	1.1
27	*Trans*-verbenol	1136	1131	2.4
28	Pinocarvone	1137	1137	4.0
29	*Cis*-pinocamphone	1149	1151	0.8
30	Cryptone	1160	1159	0.2
31	Terpinen-4-ol	1164	1163	**5.6**
32	Myrtenal	1172	1172	5.2
33	*α*-Terpineol	1176	1174	2.0
34	Myrtenol	1178	1177	**8.3**
35	*Trans*-carveol	1200	1199	0.6
36	Cuminaldehyde	1213	1216	0.3
37	Carvone	1214	1224	0.3
38	Piperitone	1226	1234	tr
39	Perylaldehyde	1260	1242	0.1
40	*α*-Terpinen-7-al	1255	1257	0.1
41	Lyratyl acetate	1264	1261	0.1
42	Perillyl alcool	1280	1275	0.1
43	Carvacrol	1278	1281	0.1
44	Germacrene D	1479	1476	0.1
45	*δ*-Cadinene	1507	1513	0.1
46	Spathulenol	1572	1565	0.1
47	Caryophyllene oxyde	1570	1568	0.1
48	Alismol^*∗*^	1619	1611	0.9
49	Bulnesol	1665	1652	0.1
	Hydrocarbon monoterpenes			29.3
	Oxygenated monoterpenes			64.7
	Hydrocarbon sesquiterpenes			0.2
	Oxygenated sesquiterpenes			1.2
	Other compounds			0.1
	Total identified (%)			95.5
	Yields (w/w vs dry material)			1.35

^a^Order of elution is given on nonpolar column (Rtx-1), ^b^retention indices of literature on the nonpolar column (lRIa) [21], ^c^retention indices on the nonpolar Rtx-1 column (RIa), and tr, trace (<0.05%). The majority compounds are indicated in bold.

**Table 3:** Antimicrobial activity of the essential oil from *X. aethiopica*.

Microorganisms	Inhibition zone of essential oil (mm)	Minimum inhibitory concentration (mg/mL)
*Staphylococcus aureus*	33.4 ± 1.8	100
*Enterococcus faecalis*	21.9 ± 1.2	>100
*Escherichia coli*	11.3 ± 0.5	>100
*Pseudomonas aeruginosa*	R	—
*Candida albicans*	22.1 ± 0.7	50

R, resistant strain; —, not tested.

## Data Availability

There are no underlying data supporting the results of this study.
